# Using stated preference methods to design gender-affirming long-acting PrEP programs for transgender and nonbinary adults

**DOI:** 10.1038/s41598-024-72920-z

**Published:** 2024-10-08

**Authors:** A. Restar, M. G. Wilson-Barthes, E. Dusic, D. Operario, O. Galárraga

**Affiliations:** 1 Departments of Epidemiology, and Health Systems and Population Health, University of Washington School of Public Health, Seattle, WA USA; 2 School of Public Health, Yale University, New Haven, CT USA; 3 Weitzman Institute, Moses Weitzman Health System, Washington, DC USA; 4 Department of Epidemiology, Brown University School of Public Health, Providence, RI USA; 5 Department of Behavioral, Social, and Health Education Sciences, Emory University Rollins School of Public Health, Atlanta, GA USA; 6 Department of Health Services, Policy and Practice, Brown University School of Public Health, Providence, RI USA

**Keywords:** Health care economics, Epidemiology, Health services

## Abstract

Integrating gender-affirming care with biomedical HIV prevention could help address the disproportionate HIV risk experienced by transgender and nonbinary (trans) adults. This discrete choice experiment assesses and identifies the most important programming factors influencing the decisions of trans adults to use injectable long-acting HIV pre-exposure prophylaxes (LA-PrEP). From March to April 2023 n = 366 trans adults in Washington state chose between four different choice profiles that presented hypothetical programs (each comprised of 5 attributes with 4 levels). We analyzed ranked choice responses using a mixed rank-ordered logit model for main effects. Respondents preferred to receive LA-PrEP from a gender-affirming care provider and a co-prescription for both oral and injectable hormones. Trans adults strongly favored 12-month protection and injection in the upper arm. No strong preferences emerged surrounding the type of health facility offering the gender-affirming LA-PrEP program. Our findings show that integrating and leveraging gender-affirming health systems, inclusive of medical services such as hormone therapy, with HIV biomedical products like LA-PrEP is strongly preferred and influential to trans adults’ decision to use LA-PrEP. Leveraging choice-based design experiments provides informative results for optimizing gender-affirming LA-PrEP programming tailored to trans adults.

## Introduction

Transgender and nonbinary (trans) adults represent 1.4 million adults in the United States (US) and experience disproportionate risk for HIV acquisition and poor HIV-related care outcomes^1,2^. While the overall HIV prevalence in the US general population is less than 0.5%, it is concentrated and reported to be 14.1% among trans women and 3.2% among trans men in the US^2^. By race/ethnicity, HIV prevalence is estimated to be 44% in Black trans women and 26% in Latina trans women^2^. This high HIV prevalence is compounded by the high proportion of undiagnosed HIV infection (i.e., > 20%) in this population^3^. Informed by this epidemiology, researchers have called for gender-inclusive intervention approaches that enable the delivery of HIV prevention tools while recognizing and supporting the health needs and gender goals of trans people^4–6^.

Recently, substantial scientific progress has been made in the development, testing, and approval of new modalities for HIV pre-exposure prophylaxes (PrEP), including long-acting injectables (LA-PrEP)^7–14^—which hold enormous potential to avert new HIV infections among trans adults. The CDC has identified trans people as a key population for PrEP, and encourages healthcare providers to discuss PrEP with trans patients as one of the key options for HIV prevention^15^. However, PrEP uptake is persistently low among trans individuals; among more than 1600 HIV-negative transgender women who responded to the National HIV Behavioral Surveillance survey, PrEP utilization rates were 35% in 2017 and 32% in 2021^16^. While reasons behind the low PrEP uptake among trans individuals include social and structural barriers such as gender-based stigma, lack of trust in healthcare providers, being susceptible to socio-economic vulnerabilities (e.g., unemployment, uninsured), and avoiding stigmatizing healthcare systems, there is limited evidence on strategies that effectively enhance PrEP uptake in this specific population.

Behavioral insights into how individuals make decisions about their HIV prevention behaviors can guide effective intervention design and implementation^17,18^. In many contexts, especially resource-limited settings, subsidies or financial incentives are used to overcome the economic and logistical barriers that threaten uptake of and adherence to HIV prevention services^19–21^. However non-financial nudges are also effective, and potentially more cost-effective, for HIV prevention^20^. Nudges based on “convenience”, “user-centeredness”, and “choice architecture” aim to structure options in a way that makes positive health decisions easy, attractive, social, and timely for the end user^22,23^. For the choice architecture of an HIV prevention program to meet these criteria, we must first understand what matters most to the program’s target participants.

Discrete choice experiments (DCEs) offer one systematic way of measuring users’ preferences. Stemming from marketing science, DCEs have become increasingly utilized in the fields of health economics and health psychology to understand individual and institutional preferences for health interventions^24^, including for HIV prevention^25,26^. Systematic reviews of DCEs eliciting preferences for PrEP cite dosing frequency, cost, drug effectiveness, and side effects to be some of the most important attributes influencing decisions around PrEP uptake and adherence^25,26^. To the best of our knowledge, however, none of these PrEP-focused DCEs have been administered to trans populations, or included attributes relevant to the gender-affirming care needs of this population. Actual uptake of LA-PrEP among trans populations is more likely to improve and sustain over time if we first uncover and quantify the stated preferences of trans adults. Understating these preferences can nudge trans populations to engage in programs that are user-centered and user-informed, thereby maximizing LA-PrEP adherence. In this analysis we aimed to quantify trans adults’ relative preferences for injectable LA-PrEP protection duration, provider type, service location, and hormone co-prescription type offered as part of a hypothetical gender-affirming, HIV prevention program.

## Methods

The discrete choice experiment (DCE) described in this paper was nested within a larger web-based survey conducted by the Washington Priority Assessment in Trans Health (PATH) Project, a community-informed statewide cross-section needs assessment study developed for, by, and with trans communities in Washington State. The exploratory DCE was conducted from a patient or client perspective, and aimed to answer the research question of “What clinical and programmatic components matter most to trans adults who may be eligible for a LA-PrEP and gender-affirming care program?”

### Attributes and levels

The attributes and levels for use in the DCE were selected by study investigators based on a review of the literature and reiterative consultations with the study’s all-trans Transgender Scientist and Stakeholder Advisory Board (TSSAB). The TSSAB, which is comprised of transgender and nonbinary scholars, leaders, and community members in Washington, was purposefully assembled to guide and review the study procedures, materials, and content, including deciding which attributes and levels are most relevant to test. Specifically, after conducting a brief review of the literature, consultations with TSSAB members were conducted reiteratively prior to survey administration to narrow the most salient attribute and possible levels that are based on realistic contexts of delivering PrEP within the Washington healthcare system. During consultations, other possible attributes and levels were also considered. Cost/affordability and support services were originally considered, but ultimately, the TSSAB decided not to include them as both attributes did not fit the study’s main purpose, which is centered on incentivizing PrEP uptake through promoting it as a free, readily available, and accessible product.

The final discrete choice experiment consisted of five attributes that contained four levels each inclusive of an opt out option (Table 1). Attributes included: “location” of LA-PrEP injection (with levels of Upper arm, Thigh, Gluteal, or No injection (opt-out)); “duration” of LA-PrEP protection against HIV infection (with levels of 12 months, 6 months, 2 months, or 0 months (opt-out)); “provider type” for administering LA-PrEP (with levels of HIV care provider, Primary care provider, Gender-affirming care provider, or No provider (opt-out)); “site” where LA-PrEP program is offered (HIV care clinic, Primary care clinic, Gender-affirming care clinic, or No site (opt-out)); and type of gender-affirming “hormone” delivered at LA-PrEP appointment (Injectable and oral hormone co-prescription, Injectable hormone co-prescription, Oral hormone co-prescription, or No co-prescription (opt-out)).

**Table 1:** Attributes and levels included in the discrete choice experiment (DCE).

Attribute	Attribute description	Number of levels	Level description
Location	Location on the body where LA-PrEP will be injected	4	Upper arm (L3)
			Thigh (L2)
			Gluteal (L1)
			No injection (opt-out) (L0) (omitted category)
Duration	Length of time for which the injectable LA-PrEP dosage would provide protection against HIV infection	4	12 months (L3)
			6 months (L2)
			2 months (L1)
			0 months (opt-out) (L0) (omitted category)
Provider type	Type of care provider administering LA-PrEP	4	HIV care provider (L3)
			Primary care provider (L2)
			Gender-affirming care provider (L1)
			No provider (opt-out) (L0) (omitted category)
Site	Site where LA-PrEP program is offered	4	HIV care clinic (L3)
			Primary care clinic (L2)
			Gender-affirming care clinic (L1)
			No site (opt-out) (L0) (omitted category)
Hormone	Type of gender-affirming hormone prescription given at LA-PrEP appointment	4	Injectable and oral hormone co-prescription (L3)
			Injectable hormone co-prescription (L2)
			Oral hormone co-prescription (L1)
			No co-prescription (opt-out) (L0) (omitted category)

Each choice profile asked respondents to choose between hypothetical programs that would require clients to come back to receive services every 2 months.

*LA-PrEP* long-acting injectable pre-exposure prophylaxis.

Concerning the levels for the duration attribute, we included levels that reflect the duration of protection from LA-PrEP that is currently available on the market, as well as aspirational protection durations. Specifically, the 2-month duration of protection level reflects long-acting cabotegravir (CAB-LA), which is an intramuscular injectable, long-acting form, with the first 2 injections administered 4 weeks apart, followed thereafter by an injection every 8 weeks^27^. The 6-month duration of protection level reflects Gilead Sciences’ long-acting capsid inhibitor, lenacapavir (sold as Sunlenca), which is administered by subcutaneous injection every six months^28^. Lenacapavir is already approved for treatment-experienced people with multidrug-resistant HIV and it is being evaluated as a twice-yearly PrEP option^28^. The 12-month duration of protection level was included by the TSSAB as an aspirational level to assist with more clearly understanding how strongly longer protection duration was preferred over other levels of this attribute, and over other attributes.

### Choice tasks and experimental design

We developed 40 choice tasks and blocked them into 4 survey versions with 10 choice tasks for each. Each block represented a separated survey, and participants were randomly assigned to one of the four survey versions and to respond to 10 program comparisons. For each program comparison, or “choice set”, respondents were asked to choose between different choice profiles that presented hypothetical programs (Supplementary Fig. 1). Hypothetical programs provided a combination of LA-PrEP and gender-affirming services every two months. Four choice profiles (each comprised of 5 attributes with 4 levels) were combined to form a choice set such that for each choice set respondents chose from random combinations of: Program A, Program B, Program C, or No Program. Each profile presented all attributes and levels being considered in the study (a full profile). We chose to include an opt-out (No Program) option for each choice task because having the option to abstain from an elective program is reflective of a respondent’s real-life choices, and opt-out options have shown to perform well in statistical analyses^29,30^. The design follows recommendations from the literature that respondents can reasonably respond to 9–16 choice sets with 4–6 attributes before reaching cognitive overload^31,32^. We used a best-best elicitation method^33,34^ where respondents were first asked to select their first choice from the 4 choice profiles (Program A, Program B, Program C, or No Program), and to then select their second-best option from the remaining 3 profiles.

Administering a full factorial design (i.e., S = 4^5^ = 1024 possible scenarios) was not possible without placing an undue burden on participants. To narrow the number of choice options while ensuring statistical efficiency, we implemented a blocked D-efficient partial factorial design using the Stata SE (College Station, TX, version 17) dcreate procedure^35^, which maximizes design efficiency based on the covariance matrix of the conditional logit model^36^. The variance–covariance matrix of the *β* coefficients assumed zero priors and two potential interactions (between duration of protection and injection site, and between provider type and hormone co-prescription type). 

We calculated our minimum required sample size as *N* > (500*c*)/(*t* × *a*), where *t* is the number of choice sets, *a* is the number of alternatives per set, and *c* is the largest number of levels for any one attribute^32^. Thus, our minimum required sample size was *N* > (500 × 4)/(10 × 4) = 50. We enrolled considerably more respondents to allow further analysis with covariates and interactions, with a final sample size of *N* = 366.

### Preference elicitation and data collection

The parent PATH study used convenience sampling to recruit participants, which included reaching out to local organizations, community listservs, social media platforms, trans-focused support groups, and events. To be eligible, individuals had to be 18 years or older, identify as transgender and/or nonbinary, currently live in Washington State, and be willing and able to provide electronic consent to participate in the study. Once screened for eligibility and enrolled, participants were directed to the web-based survey, which was designed to be user-friendly (e.g., simple design and wording, preventing multiple responses, allowing for saving and finishing later). The survey captured participants’ demographic information, and healthcare experiences and needs, alongside the discrete choice experiment (DCE) that surveyed LA-PrEP programming preferences. The entire survey was piloted to take less than 25 min to complete, with the DCE portion taking approximately 10-min to complete.

Data collection occurred from March to April of 2023. All research procedures were reviewed and approved by the University of Washington Human Subjects Division (IRB #:STUDY00015878) in accordance with the Declaration of Helsinki. Informed consent was obtained from all participants.

### Statistical analyses

We first used a conditional logit regression to explore patients’ primary (first) choice of a hypothetical program. In a conditional logit, the probability of choosing among at least two alternatives is related to the attribute levels characterizing the alternatives^37,38^. The dependent variable was the participants’ first preferred program in each choice task and the independent variables were the dummy-coded levels of each of the attributes in each profile. Dummy coding estimates the preference weights of a given attribute level relative to the omitted level of that attribute^37,38^.

To assess main effects, we then used the best-best approach with an ‘exploded logit’ by expanding the data from each choice set into two choice subsets. The best-best approach elicits a respondent’s first-best choice of all hypothetical programs and then asks for their second-best choice from the remaining alternatives^39^. Compared to a traditional best–worst DCE design, the best-best approach has shown to improve efficiency of data collection (by eliciting additional observations per choice and reducing respondent burden with fewer choice sets) as well as statistical efficiency^34^. The first subset contained four rows of data representing the four alternatives in the original set where the dependent variable (choice) = 1 for the first-best and = 0 for the remaining alternatives. The second subset identified the second-best from the remaining three alternatives^39,40^. We analyzed the ranked choice responses using a mixed rank-ordered logit (MROL) model, which included normally distributed random parameters to allow for unobserved heterogeneity in latent preferences for a chosen Program compared with No Program. We included a constant term; age, gender, race, highest education completed, income before taxes last year, and geography of current residence were modelled as continuous or categorical variables; other attributes (has ever taken PrEP, has ever engaged in sex work) were dummy coded. Specifications of the MROL model are included in the supplementary material.

The MROL model showed the best performance based on the Akaike information criterion (AIC) values, and exploited more of the available data, and thus was used to assess main results. We used a rank ordered logit (ROL) model and a generalized multinomial logit model (GMNL), additionally, to check for robustness of main results. Lastly, to explore preference heterogeneity, we looked at the average program choice probabilities over the entire range of participant ages, income, and education levels.

## Results

### Respondent characteristics

Table 2 shows the characteristics of the 366 trans participants who completed the DCE. Respondents were 29 years of age on average (IQR: 26–32 years, not shown). Most were women (79.8%), White (64.2%), lived in or near a large city (59.6%), had health insurance (99.5%), and reported completing 1–3 years of college or less (88.8%). Only 16.4% (i.e., sixty participants) reported having ever previously taken PrEP for HIV prevention.

**Table 2:** Characteristics of N = 366 survey respondents.

	(1)	(2)	(3)
	Mean (SD) or % (N)^a^	Minimum	Maximum
Gender identity		1	5
Woman, including transgender woman	79.8% (292)		
Man, including transgender man	9.0% (33)		
Nonbinary, including gender nonconforming, genderqueer	10.4% (38)		
Two-spirit	0.8% (3)		
Sex assigned at birth		1	3
Male	82.5% (302)		
Female	17.2% (63)		
Intersex	0.3% (1)		
Age at survey (years)	29.02 (4.68)	18	60
Age at survey squared	864.12	324	3600
Race		1	8
Asian/Asian American	11.2% (41)		
Black/AA	11.5% (42)		
Hispanic, Latino, Spanish origin	8.5% (31)		
Middle Eastern/North African	1.1% (4)		
Native Hawaiian/Pacific Islander	0.8% (3)		
White	64.2% (235)		
American Indian	1.4% (5)		
Other	1.1% (4)		
Missing	0.3% (1)		
Currently covered by a health insurance plan^b^		0	1
No	0% (0)		
Yes	99.5% (364)		
Missing	0.5% (2)		
Ever taken PrEP		0	1
No	81.7% (299)		
Yes	16.4% (60)		
Missing	1.9% (7)		
Ever engage in sex work		0	1
No	92.1% (337)		
Yes	6.3% (23)		
Missing	1.6% (6)		
Geography of current residential community		1	4
Rural area	13.4% (49)		
Small city or town	27.0% (99)		
Large city	39.1% (143)		
Suburb near a large city	20.5% (75)		
Highest education completed^c^		2	7
Grades 1 through 8 (Elementary and/or middle school)	0.3% (1)		
Grades 9 through 12 (Some high school)	21.9% (80)		
Grade 12 or GED (High school graduate)	29.8% (109)		
College 1 year to 3 years (Some college or technical school)	36.9% (135)		
College 4 years or more (College graduate)	7.9% (29)		
Graduate school	3.3% (12)		
Personal income (before taxes) last year		1	8
No income	1.1% (4)		
Less than $14,999	5.2% (19)		
$15,000–$29,999	7.4% (27)		
$30,000–$49,999	43.4% (159)		
$50,000–$74,999	36.3% (133)		
$75,000–$99,999	2.5% (9)		
$100,000–$149,999	0.5% (2)		
$150,000 or more	0.8% (3)		
Missing	2.7% (10)		

^a^Data are presented as mean (SD) for continuous measures, and % (n) for categorical measures.

^b^Survey question asked “Are you currently covered by any of the following types of health insurance or health coverage plans? (Check all that apply)”, where respondents were considered to have insurance if they selected at least one of the following providers Medicaid/Apple Care, Medicare, Veteran’s Administration, Private/Work/School insurance (e.g. Blue Cross), Parent’s insurance.

^c^Response options for “What is the highest education you’ve attained?” additionally included “Never attended school (1)”. However, no respondents selected this category.

### Attribute and program preferences

**Fig. 1:**
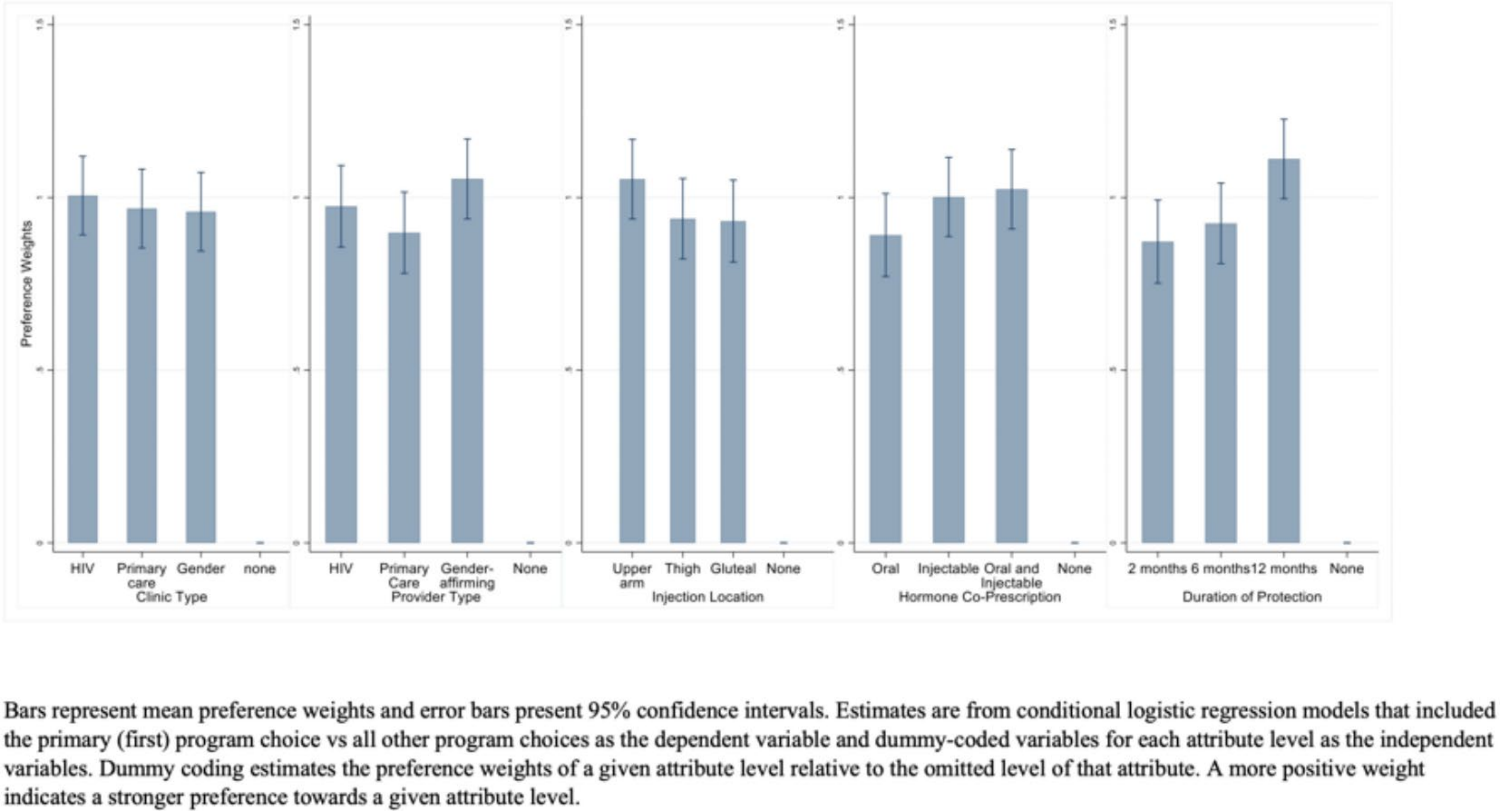
Mean preference weights with 95% confidence intervals (CIs). Bars represents mean preference weights and error bars present 95% confidence intervals. Estimates are from conditional logistic regression models that included the primary (first) program choice vs all other program choices as the dependent variable and dummy-coded variables for each attribute level as the independent variables. Dummy coding estimates the preference weights of a given attribute level relative to the omitted level of that attribute. A more positive weight indicates a stronger preference towards a given attribute level.

Figure 1 and Supplementary Table 1 show preference weights for the five attributes based on the primary (first) choice from the conditional logit regression model. Survey respondents preferred receiving LA-PrEP from a gender-affirming care provider (rather than an HIV provider or primary care physician), and preferred receiving a co-prescription for oral and injectable gender-affirming hormones (rather than solely oral or injectable) at their LA-PrEP visit. Respondents strongly preferred LA-PrEP that provided protection against HIV for up to 12 months, and being administered the LA-PrEP injection in their upper arm (rather than in the glutes or thigh). There was no strong preference as to the type of site that should offer the gender-affirming LA-PrEP program. As shown in Table 3, when normalized to the scale of 100%, the duration of LA-PrEP protection had the highest relative attribute importance (37.1%), followed by injection site on the body where LA-PrEP is administered (21.4%) and type of provider administering LA-PrEP (19.9%). The type of health facility offering the LA-PrEP program had the lowest relative importance (3.2%).

**Table 3:** Relative importance of the 5 DCE attributes.

Attributes	Difference in preference weights	Relative importance (%)
Location where LA-PrEP program is offered	0.021	3.2
Duration of LA-PrEP protection against HIV	0.249	37.1
Type of care provider administering LA-PrEP	0.134	19.9
Injection site on body where LA-PrEP is administered	0.144	21.4
Type of gender-affirming hormone co-prescription given at LA-PrEP appointment	0.124	18.4

The relative importance of each attribute was calculated as the difference between the maximum and minimum preference weights for each attribute from effects-coded conditional logit models, divided by the sum of the differences in all five attributes.

*LA-PrEP* long-acting injectable pre-exposure prophylaxis.

Table 4 shows results from the MROL model using the complete ranking data coming from the best–best choices of DCE respondents. In terms of alternative-specific variables, the coefficient on the 12-month duration of LA-PrEP protection is positive (β = 0.127) and strongly significant (*p* < 0.001), indicating that the probability of choosing a hypothetical program increases as the length of LA-PrEP protection against HIV increases. Similarly, the coefficients on LA-PrEP being administered in the upper arm (β = 0.073), on LA-PrEP being administered by a gender-affirming provider (β = 0.087), and LA-PrEP visits also providing a co-prescription for oral and injectable hormones (β = 0.10) are also positive and statistically significant (*p* < 0.05). Although not statistically significant, the coefficients for the program site attribute levels suggest that trans adults may prefer to attend a gender-affirming LA-PrEP program at a gender clinic rather than at an HIV clinic or primary care facility. The standard deviations (column 2) are large relative to the means of the associated attribute coefficients (column 1), which indicates there is heterogeneity in preferences across respondents. Supplementary Table 2 shows the results of the rank ordered logit and generalized multinomial logit models, which show generally consistent trends as the MROL model for the relevant alternative-specific coefficients.

**Table 4:** Results of mixed rank-ordered logit (MROL) model^d^.

	(1)	(2)	(3)	(4)	(5)
	MROL—Coeff^a^	MRO—SD^b^	MROL—coeff program A^c^	MROL—coeff program B^c^	MROL—coeff program C^c^
Alternative-specific variables					
Site of LA-PrEP injection is arm (= 1)	0.0729**	0.0224			
	(0.0350)	(0.133)			
Duration of LA-PrEP protection against HIV is 12 months (= 1)	0.127***	0.531***			
	(0.0478)	(0.0860)			
Provider administering LA-PrEP is a gender-affirming provider (= 1)	0.0865**	0.128			
	(0.0399)	(0.219)			
Hormone co-prescription given at LA-PrEP visit is for oral and injectable hormones (= 1)	0.0993***	0.144			
	(0.0355)	(0.0948)			
Site of LA-PrEP program delivery is HIV clinic (= 1)	− 0.00902	0			
	(0.0370)	(0)			
Site of LA-PrEP program delivery is gender clinic (= 1)	0.0522 (0.0368)	0.0958 (0.1041)			
Case-specific variables					
Age			− 0.0699***	− 0.0733***	− 0.0515***
			(0.0179)	(0.0199)	(0.0154)
Gender identity			0.702**	0.684**	0.747**
			(0.305)	(0.301)	(0.302)
Sex assigned at birth			− 0.119	− 0.435	− 0.477
			(0.423)	(0.412)	(0.407)
Race			0.104***	0.0849***	0.0410*
			(0.0264)	(0.0240)	(0.0247)
Level of education			0.145**	0.160**	0.134**
			(0.0654)	(0.0645)	(0.0619)
Annual income			− 0.145	− 0.0179	− 0.0360
			(0.0947)	(0.0900)	(0.0732)
Ever taken PrEP (= 1)			1.299***	1.433***	1.007***
			(0.325)	(0.349)	(0.326)
Ever engaged in sex work (= 1)			− 0.277	0.111	0.131
			(0.415)	(0.388)	(0.398)
Geography of current residential community			0.125**	0.0415	0.128***
			(0.0507)	(0.0448)	(0.0468)
Constant			0.962	1.676**	1.063
			− 0.0699***	− 0.0733***	− 0.0515***
Observations	24,220				
Log-likelihood	− 7769				
AIC	15619.61				
BIC	15900.14				
Parameters	42				

^a^Means of random coefficients for alternative-specific variables.

^b^Standard deviation (SD) of the random coefficients for alternative-specific variables.

^c^Using No Program/Opt-Out as reference. A positive (negative) sign for an attribute means that level impacted positively (negatively) on utility and thus increased (reduced) the probability of choosing an alternative with that level.

^d^Number of participants = 366.

Robust standard errors in (parentheses) ***p < 0.01, **p < 0.05, *p < 0.1.

**Fig. 2:**
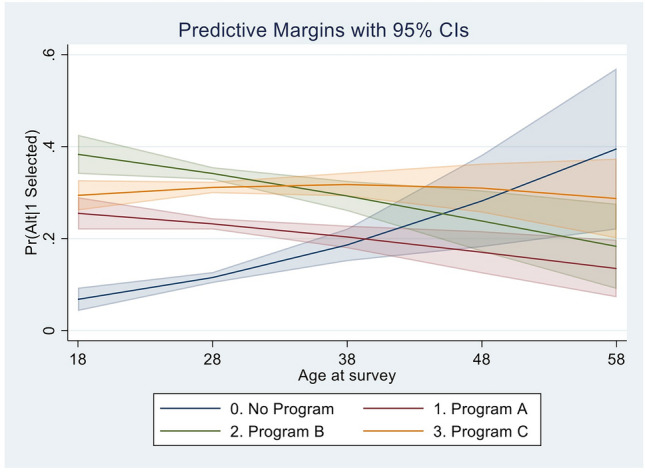
Average choiced probabilities over the entire age range. The figure presents the averaged choice probabilities over the entire age range, excluding all other case-specific variables from the rank order logit model. Among individuals between 18 and 35 years of age, all three hypothetical programs have a 5–25% higher probability of being chosen than the opt-out option, depending on age and program type. For participants 50 years and older, the averaged probability of choosing the opt out option is higher than the probability of choosing any hypothetical program.

Figure 2 shows the averaged choice probabilities over the entire age range of DCE respondents. Among individuals between 18 and 35 years of age, all three hypothetical programs have a 5 – 25 percentage-point higher probability of being chosen than the opt-out option, depending on age and program. After the age of 50, the average probability of choosing the opt-out option (i.e., no program) is higher than the probability of choosing any hypothetical program. No clear trend in program choice probability was seen over the range of possible annual income; and the probability of choosing a hypothetical program over no program did not seem to vary by education level (Supplementary Fig. 3a and b).

### Robustness tests

Estimates from additional models are presented in Supplementary Table 2. First, in columns (1–4) is the ROL model, which shows similar results as the main model. Column (5) is the GMNL, which also shows similar results as the MROL except for site of LA-PrEP delivery being HIV clinic or gender clinic, which are both positive and significant (*p* < 0.01). The τ parameter for the GMNL was significant, which indicates unobserved heterogeneity.

Supplementary Table 3 presents the correlation matrix used to check for orthogonality. The pairwise correlation coefficients are generally low, which provides evidence that each relevant attribute level and covariate vary independently of one another.

## Discussion

The findings from this study provide valuable insights into the attributes and program preferences of trans adults regarding LA-PrEP for HIV prevention. The results indicate that trans adults overwhelmingly prefer to receive LA-PrEP within a gender-affirming healthcare approach^41^– that is, situating LA-PrEP continuum services within a health system that also recognizes and ensures trans adults’ gender goals are medically, socially, and structurally supported^42,43^. The directions of the findings support what most researchers have persistently emphasized when designing gender-affirmative public health programming for, by, and with trans communities^44,45^. We further add empirical evidence, by using a DCE design for health programming, so that researchers and program planners can identify and optimize specific attributes and program features that are influential and deemed most important among transgender adults. Making the programs and services more convenient and responsive to the users’ preferences and needs is a crucial tenet of the behavioral economics’ “choice architecture” concept^46^.

We found that respondents showed a strong preference for receiving LA-PrEP from a gender-affirming care provider rather than an HIV provider or primary care physician, and that the type of health facility offering the gender-affirming LA-PrEP program did not significantly influence preferences (under two of the three main specifications). These findings underscore two important implications for LA-PrEP programming: First, it highlights the importance of culturally competent gender-affirming healthcare systems and the need for providers who are knowledgeable about trans patients’ health needs. Given that trans individuals often face unique healthcare challenges (e.g., being denied health insurance coverage) and avoid healthcare visits due to past negative experiences (e.g., experiencing mistreatment and/or being stigmatized from providers and staff)^47–49^, having providers who understand and respect trans people’s gender identity can greatly enhance their overall healthcare experience. Second, while gender-affirming care providers are likely more adept to providing such an approach to care compared to HIV providers or primary care physicians (and therefore most preferred by trans adults), the results also indicate that trans adults are open to accessing these services in any type of healthcare settings, as long as the care is affirming and meets their specific needs. These two findings, therefore, highlight the importance of situating LA-PrEP in healthcare environments that are welcoming and inclusive with providers that practice gender-affirming approaches to care, regardless of the specific type of facility. Healthcare providers and public health professionals, PrEP programmers, and policymakers should work towards ensuring that gender-affirming care is available and accessible across different healthcare settings when scaling up LA-PrEP.

Additionally, we also found that trans adults prefer having a co-prescription for both oral and injectable gender-affirming hormones during the LA-PrEP visit, highlighting the desire for comprehensive and integrated healthcare services, particularly between HIV prevention and gender-affirming medical care. While gender-affirming hormone therapy is a priority for many trans populations, understanding ways to address trans people’s concerns and/or misinformation regarding adverse side effects or drug interactions of PrEP with co-prescribed medicines like hormones via an intervention is vital to increasing PrEP uptake and its programming^50–52^. To combat misinformation, for example, the Center for Disease Control and Prevention (CDC) recommends delivering evidence-based messaging given that there are no known or predicted drug interactions between the medications used for PrEP and gender-affirming hormones^53^. This finding suggests that trans individuals value the convenience and holistic approach of receiving multiple aspects of their care in a single visit, which is similar to what other studies calling for multilevel component intervention design have noted previously^41,54,55^. By combining LA-PrEP with gender-affirming hormone therapy, healthcare providers can offer a more comprehensive and personalized approach to trans health, particularly meeting both HIV prevention needs and gender goals of trans individuals.

Respondents also expressed a strong preference for LA-PrEP that provides protection against HIV for up to 12 months. This longer duration of protection aligns with the desire for a simplified and less frequent dosing regimen^56–58^, which can enhance adherence and reduce the burden of medication management. Trans individuals may already have complex medication regimens, and having a longer-acting form of HIV prevention can alleviate some of the challenges associated with adherence to daily medication^59,60^. Moreover, the location of the LA-PrEP injection site was another important factor influencing preferences. Respondents favored receiving the injection in their upper arm rather than in the glutes or thigh. This preference may be driven by factors such as comfort, ease of self-administration, and potential visibility of injection sites^56–58^ Understanding these preferences can inform the development of administration techniques and training materials for healthcare providers to ensure that LA-PrEP injections are delivered in a manner that aligns with the preferences and needs of transgender individuals.

While this study provides valuable insights, it is not without limitations. The sample primarily consisted of trans adults from the state of Washington, which may limit the generalizability of the findings to other geographical settings. Future research should aim to include a more diverse sample to capture a broader range of perspectives. Additionally, longitudinal studies can provide further insights into how preferences may change over time and in response to different biomedical products that are or will be in the pipeline. Moreover, this study focused on preferences for LA-PrEP attributes and programs. Future research could explore other factors that may influence decision-making, such as cost (including subsidies or incentives), accessibility, and side effects, which can also be identified and tested via a DCE design^61–65^. In terms of the experimental design, using a partial factorial design (as was done in this DCE) can make it difficult to distinguish interaction effects from main effects, which can lead to incorrect interpretation of findings if or when confounded effects are substantial. Also, deciding upon the second-best choice in a best-best preference elicitation design may be mentally difficult for respondents in some instances^34^. Lastly, while it is not a limitation of the study per se, it is important to note that there was heterogeneity in preferences across respondents, as indicated by the large standard deviations relative to the means of the attribute coefficients. While results are generally significant at the group level, this highlights the diversity within the trans population and the need for more research to identify within-group patterns to inform personalized approaches to care and service delivery. Healthcare providers should be mindful of this heterogeneity and strive to provide individualized care that takes into account the unique preferences and needs of each transgender individual.

## Conclusion

Overall, these findings contribute to a better understanding of the preferences of trans adults regarding LA-PrEP for HIV prevention. The insights gained from this study can inform the development and implementation of gender-affirming healthcare programs and interventions aimed at improving HIV prevention and care outcomes among transgender individuals. By incorporating these preferences into program design and implementation, healthcare providers and policymakers can better meet the needs of transgender individuals and improve health outcomes in this population. Continued research and collaboration are needed to further contextualize how LA-PrEP fits into the lives and geographical contexts of trans adults and approaches to ensure equitable access to new HIV biomedical products for trans communities. The use of a behavioral economics general approach in conjunction with a DCE design can be a useful tool to identify and optimize ways to address the unique healthcare needs of trans individuals and to provide the affirming and comprehensive programming care this population deserves.

## Supplementary Information

Supplementary Information.

## Data Availability

The data that support the findings of this study are available from the corresponding author upon reasonable request and approval from the University of Washington IRB.
