# Supplementary material for: Using stated preference methods to design gender-affirming long-acting PrEP programs for transgender and nonbinary adults
Source: Sci Rep. 2024 Oct 8;14:23482. doi: 10.1038/s41598-024-72920-z (PMC11461737; doi:10.1038/s41598-024-72920-z)
Supplement: Supplementary file 1 — Supplementary Information. [file 41598_2024_72920_MOESM1_ESM.docx]

**Supplementary Materials**

[**Supplementary Figure 1.** Examples of computer-based sequential screens for choice scenario simulation for discrete choice experiment (DCE). 2](#_Toc172816001)

[**Supplementary Table 1.** Mean preference weights for each dummy-coded attribute level 3](#_Toc172816002)

[**Supplementary Material.** Specifications of the mixed rank-ordered logit model 4](#_Toc172816003)

[**Supplementary Table 2.** Results of rank ordered logit (ROL) and generalized multinomial logit (GMNL) models 5](#_Toc172816004)

[**Supplementary Figure 3a.** Averaged choice probabilities over the entire range of education level 7](#_Toc172816005)

[**Supplementary Figure 3b.** Averaged choice probabilities over the entire income range 8](#_Toc172816006)

[**Supplementary Table 3.** Correlation matrix to check for orthogonality 9](#_Toc172816007)

[**References for Supplementary Material** 11](#_Toc172816008)

## **Supplementary Figure 1.** Examples of computer-based sequential screens for Best-Best choice scenario simulation for the discrete choice experiment (DCE).


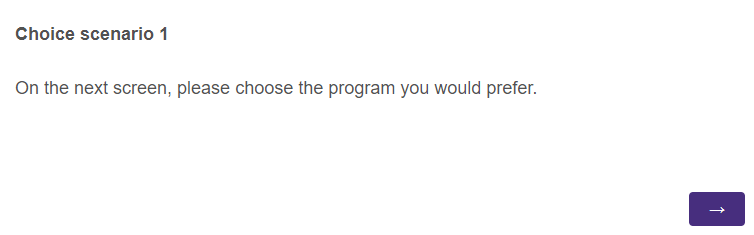


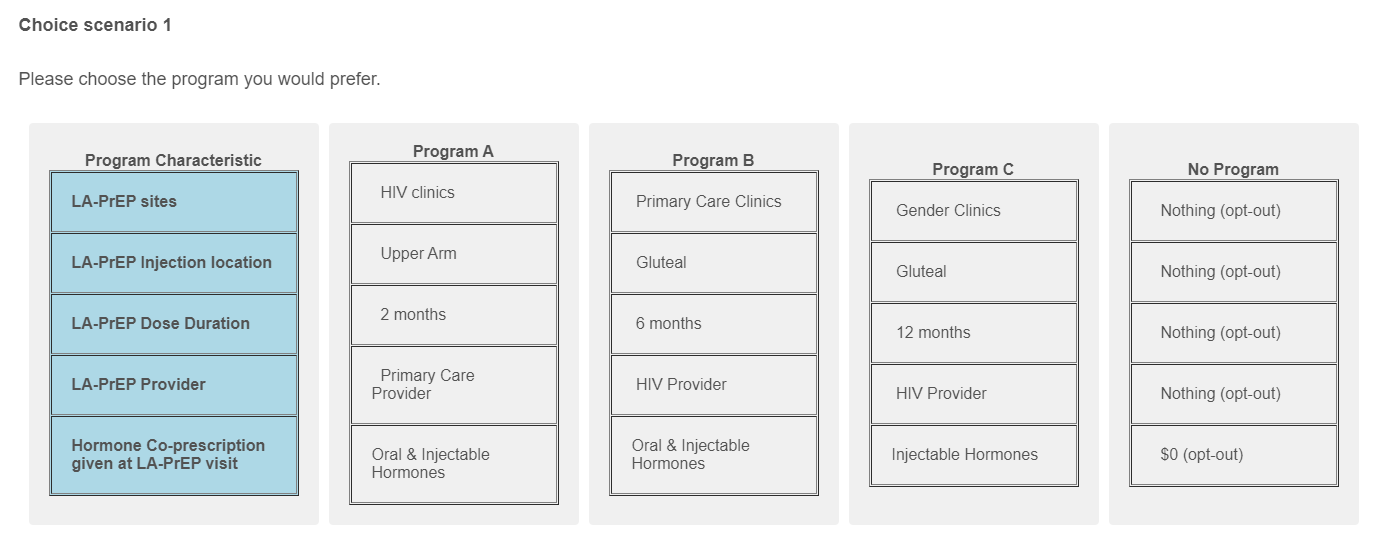


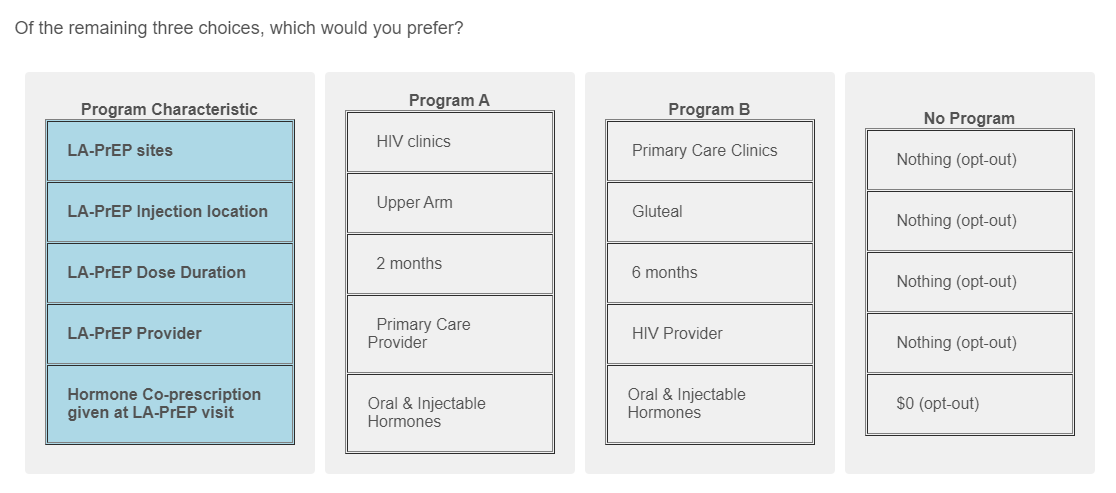


In this example, the participant chooses Program C in the Choice Scenario 1 for the first-best option; thus, Program C is no longer available to the participant when choosing the second-best option.

# **Supplementary Table 1.** Mean preference weights for each dummy-coded attribute level

| **Attributes** | **Level** | **Coefficient** | **95% CI** | |
| --- | --- | --- | --- | --- |
| Location where LA-PrEP program is offered | HIV Clinics | 1.006 | .8916078 1.119406 | |
|  | Primary care clinics | 0.968 | .8538951 1.081861 | |
|  |  |  |  | |
|  | Gender clinics | 0.959 | .8447224 1.072442 | |
|  |  |  |  | |
| Duration of LA-PrEP protection against HIV | 2 months | 0.872 | .7515843 .9924788 | |
|  | 6 months | 0.925 | .8084857 1.041669 | |
|  |  |  |  | |
|  | 12 months | 1.112 | .9962833 1.226637 | |
|  |  |  |  | |
| Type of care provider administering LA-PrEP | HIV provider | 0.974 | .8563127 1.092491 | |
|  | Primary care provider | 0.898 | .780317 1.015592 | |
|  |  |  |  | |
|  | Gender-affirming provider | 1.053 | .9378498 1.169375 | |
|  |  |  |  | |
| Injection site on body where LA-PrEP is administered | Gluteal | 0.932 | .8126708 1.05043 | |
|  | Thigh | 0.938 | .8221688 1.054679 | |
|  | Upper Arm | 1.053 | .9378333 1.168307 | |
|  |  |  |  | |
| Type of gender-affirming hormone co-prescription given at LA-PrEP appointment | Oral hormones | 0.891 | .7709104 1.011333 | |
|  |  |  |  | |
|  | Injectable hormones | 1.002 | .8869637 1.116041 | |
|  |  |  |  | |
|  | Injectable and oral hormones | 1.024 | .9087866 1.139131 | |
| LA-PrEP: Long-acting injectable pre-exposure prophylaxis  Table presents coefficients and 95% confidence intervals (CI) as graphically presented in Figure 1. Estimates are from conditional logistic regression models that included the primary (first) program choice vs all other program choices as the dependent variable and dummy-coded variables for each attribute level as the independent variables. Dummy coding estimates the preference weights of a given attribute level relative to the omitted level of that attribute. To address issues of collinearity, conditional logit models were run separately for each attribute. More positive weights indicate a stronger preference towards a given attribute level. | | | |  |

# **Supplementary Material.** Specifications of the mixed rank-ordered logit model

We followed methods similar to those implemented by Ghijben P et al. 2014^1^ and Galárraga et al. 2020^2^ to implement our mixed rank-ordered logit (MROL) model. The model elicits both the first-best and second-best preferences in each choice-set. The probability of any ranking of the alternatives from best to worst is expressed as the product of logit formulas.

$$P\left[ U_{{ij}_{A}t}>U_{{ij}_{B}t}>U_{{ij}_{C}t} \right]= \frac{e^{V_{ij_{A}t}}}{\sum_{j=A,B,C} e^{V_{ijt}}}*\frac{e^{V_{ij_{B}t}}}{\sum_{j=B,C} e^{V_{ijt}}} (Eq.1)$$

The first ratio in equation 1 is the first-best choice from the full choice-set. The second ratio in equation 1 is the second-best choice from the remaining alternatives after the first-best alternative has been removed. Conditional on *β_i_*, the probability of a respondent’s ranking simplifies to the rank-ordered logit model (Equation 1). However, since *β_i_* is unknown, the unconditional probability is the integral of this product over the density of *β*.

$$P\left[ U_{{ij}_{A}t}>U_{{ij}_{B}t}>U_{{ij}_{C}t} \right]=\int\left( \frac{e^{V_{ij_{A}t}}}{\sum_{j=A,B,C} e^{V_{ijt}}}*\frac{e^{V_{ij_{B}t}}}{\sum_{j=B,C} e^{V_{ijt}}} \right)*f\left( \beta| \theta\right)d\beta(Eq.2)$$

The ranked choice responses were analyzed using a single MROL model using Stata’s cmxtmixlogit command^3^. The model included a normally distributed random parameter to account for unobserved heterogeneity in underlying preferences for a given program compared to ‘no program’. The choice responses were modelled as the dependent variable and the difference in levels for each of the attributes were modelled as the independent variables, with ‘no program’ assumed as the base. The model included all non-collinear attributes (*X^’^_ijt_ =* *LA-PrEP injection site, duration of protection, type of provider administering LA-PrEP, type of hormone co-prescription given at LA-PrEP visit,* and *location of LA-PrEP program delivery)* as independent variables and a choice-specific constant for ‘no program’ (*ASC_NoPrg_*). The model also allowed for observed heterogeneity by including case-specific variables (age, gender, birth sex, race, education level, personal income last year, ever taken PrEP, ever engaged in sex work, geography of current residence) which varied across participants but not program alternatives. Case-specific variables were interacted with the intercept (*ASC_NoPrg_.Z_i_)* and selected treatment attributes (*X^’^_ijt_.Z_i_)* to explore the effect of participant characteristics on preferences. The MROL model can be specified as:

$$V_{\mathrm{ij}}= {ASC}_{NoPrg}+ X_{\mathrm{ijt}}^{'}\beta+X_{\mathrm{ijt}}^{'}.X_{\mathrm{ijt}}^{'}.\beta+{{ASC}_{NoPrg}.Z}_{i}^{'}\gamma+ X_{ijt}.Z_{i}^{'}\alpha(Eq.3)$$

# **Supplementary Table 2.** Results of rank ordered logit (ROL) and generalized multinomial logit (GMNL) models

|  | (1) | (2) | (3) | (4) | (5) | (6) |
| --- | --- | --- | --- | --- | --- | --- |
| VARIABLES | ROLa | ROLa – Program A^‡^ | ROLa – Program B^‡^ | ROLa –  Program C^‡^ | GMNL –  Coefficients^‡^ | GMNL  Scale heterogeneity |
| *Alternative-specific variables* |  |  |  |  |  |  |
|  |  |  |  |  |  |  |
| Site of LA-PrEP injection is arm (=1) | 0.0794** |  |  |  | 0.222*** |  |
|  | (0.0337) |  |  |  | (0.0300) |  |
| Duration of LA-PrEP protection against HIV is 12 months (=1) | 0.110*** |  |  |  | 0.513*** |  |
|  | (0.0399) |  |  |  | (0.0422) |  |
| Provider administering LA-PrEP is gender-affirming provider (=1) | 0.0740** |  |  |  | 0.201*** |  |
|  | (0.0375) |  |  |  | (0.0315) |  |
| Hormone co-prescription given at LA-PrEP visit is for oral and injectable hormones (=1) | 0.0763** |  |  |  | 0.241*** |  |
|  | (0.0347) |  |  |  | (0.0312) |  |
| Site of LA-PrEP program delivery is HIV clinic (=1) | -0.0211 |  |  |  | 0.210*** |  |
|  | (0.0362) |  |  |  | (0.0322) |  |
| Site of LA-PrEP program delivery is gender clinic (=1) | 0.0442 |  |  |  | 0.218*** |  |
|  | (0.0352) |  |  |  | (0.0311) |  |
| *Case-specific variables*^ |  |  |  |  |  |  |
|  |  |  |  |  |  |  |
| age |  | -0.0648*** | -0.0687*** | -0.0482*** |  |  |
|  |  | (0.0172) | (0.0191) | (0.0151) |  |  |
| genderid |  | 0.677** | 0.657** | 0.716** |  |  |
|  |  | (0.305) | (0.301) | (0.302) |  |  |
| birthsex |  | -0.0167 | -0.295 | -0.356 |  |  |
|  |  | (0.419) | (0.402) | (0.399) |  |  |
| race |  | 0.0955*** | 0.0745*** | 0.0402* |  |  |
|  |  | (0.0254) | (0.0229) | (0.0239) |  |  |
| education |  | 0.150** | 0.155** | 0.130** |  |  |
|  |  | (0.0639) | (0.0627) | (0.0606) |  |  |
| income |  | -0.159* | -0.0305 | -0.0518 |  |  |
|  |  | (0.0940) | (0.0899) | (0.0746) |  |  |
| everprep (=1) |  | 1.364*** | 1.527*** | 1.085*** |  |  |
|  |  | (0.329) | (0.349) | (0.331) |  |  |
| Eversexwork (=1) |  | -0.331 | 0.0245 | 0.0856 |  |  |
|  |  | (0.421) | (0.395) | (0.409) |  |  |
| livegeo |  | 0.114** | 0.0366 | 0.116** |  |  |
|  |  | -0.0648*** | -0.0687*** | -0.0482*** |  |  |
| constant |  | 0.826 | 1.531** | 0.981 |  | 0.943*** |
|  |  | (0.672) | (0.718) | (0.620) |  | (0.0825) |
| Participants | 366 | 366 | 366 | 366 | 366 | 366 |
| Observations | 24,220 | 24,220 | 24,220 | 24,220 | 25,620 | 25,620 |
| Log-likelihood | -7788 | -7788 | -7788 | -7788 | -8701 | -8701 |
| parameters | 36 | 36 | 36 | 36 | 7 | 7 |
| AIC | 15648 | 15648 | 15648 | 15648 | 17417 | 17417 |
| BIC | 15894 | 15894 | 15894 | 15894 | 17474 | 17474 |
| τ |  |  |  |  |  | 0.9433 (0.0825)*** |

*** p<0.01, ** p<0.05, * p<0.1

Table presents coefficients and robust standard errors clustered at individual level in (parentheses).

LA-PrEP=Long-acting injectable pre-exposure prophylaxis; ROL=rank ordered logit estimated via a conditional logit regression [McFadden’s choice model]); GMNL=generalized multinomial logit; AIC=Akaike information criterion; BIC=Bayesian information criterion.

‡ Using Opt-Out / No Program as reference. A positive (negative) sign for an attribute means that level impacted positively (negatively) on utility and thus increased (reduced) the probability of choosing an alternative with that level.

^ In the estimation, all case-specific variables were modelled as continuous, with the exception of having ever taken PrEP which was dummy coded (=1 if yes).

# **Supplementary Figure 3a.** Averaged choice probabilities over the entire range of education level

The figure presents the averaged choice probabilities over the entire range of education level, excluding all other case-specific variables from the rank order logit model. All three hypothetical programs have a 5% to 35% higher probability of being chosen than the opt-out option. The averaged probability of choosing any hypothetical is always higher than the probability of choosing the opt out option, regardless of respondent’s education level.

# **Supplementary Figure 3b.** Averaged choice probabilities over the entire income range

The figure presents the averaged choice probabilities over the entire range of annual income, excluding all other case-specific variables from the rank order logit model. Among individuals between earning less than $100,000 per year, all three hypothetical programs have a 2 – 24% higher probability of being chosen than the opt-out option, depending on income and program type. For participants earning at least $100,000 per year, the averaged probability of choosing the opt out option over any hypothetical program begins to increase.

# **Supplementary Table 3.** Correlation matrix to check for orthogonality

|  | Site of LA-PrEP injection is arm (=1) | Duration of LA-PrEP protection against HIV is 12 months (=1) | Provider administering LA-PrEP is a gender-affirming provider (=1) | Hormone co-prescription given at LA-PrEP visit is for oral and injectable hormones (=1) | Site of LA-PrEP program delivery is HIV clinic (=1) | Site of LA-PrEP program delivery is gender clinic (=1) | age | genderid | birthsex | race | education | income | everprep (=1) | eversexwork (=1) | livegeo |
| --- | --- | --- | --- | --- | --- | --- | --- | --- | --- | --- | --- | --- | --- | --- | --- |
| Site of LA-PrEP injection is arm (=1) | 1 |  |  |  |  |  |  |  |  |  |  |  |  |  |  |
| Duration of LA-PrEP protection against HIV is 12 months (=1) | 0.105*** | 1 |  |  |  |  |  |  |  |  |  |  |  |  |  |
| Provider administering LA-PrEP is a gender-affirming provider (=1) | 0.152*** | 0.181*** | 1 |  |  |  |  |  |  |  |  |  |  |  |  |
| Hormone co-prescription given at LA-PrEP visit is for oral and injectable hormones (=1) | 0.0951*** | 0.107*** | 0.110*** | 1 |  |  |  |  |  |  |  |  |  |  |  |
| Site of LA-PrEP program delivery is HIV clinic (=1) | 0.117*** | 0.136*** | 0.169*** | 0.153*** | 1 |  |  |  |  |  |  |  |  |  |  |
| Site of LA-PrEP program delivery is gender clinic (=1) | 0.118*** | 0.0927*** | 0.105*** | 0.120*** | -0.321*** | 1 |  |  |  |  |  |  |  |  |  |
| age | 0.00416 | 0.00539 | 0.00939 | 0.00467 | 0.00283 | 0.00535 | 1 |  |  |  |  |  |  |  |  |
| genderid | -0.00418 | -0.0120 | -0.00445 | -0.0148* | 0.00381 | -0.0119 | -0.229*** | 1 |  |  |  |  |  |  |  |
| birthsex | -0.00262 | -0.0110 | 0.00266 | -0.0151* | 0.00553 | -0.00964 | -0.176*** | 0.787*** | 1 |  |  |  |  |  |  |
| race | -0.000483 | -0.00678 | -0.00489 | -0.00252 | 0.000152 | -0.00208 | -0.0666*** | 0.103*** | 0.125*** | 1 |  |  |  |  |  |
| education | 0.000583 | -0.00934 | -0.00428 | -0.00789 | 0.00539 | -0.00872 | -0.0204** | 0.408*** | 0.423*** | 0.0689*** | 1 |  |  |  |  |
| income | 0.00352 | 0.00536 | -0.000504 | -0.00269 | -0.00238 | 0.00160 | 0.364*** | -0.155*** | -0.218*** | -0.0601*** | -0.0645*** | 1 |  |  |  |
| everprep (=1) | -0.00663 | -0.0168** | -0.00561 | -0.00336 | -0.0123 | -0.00102 | -0.112*** | 0.194*** | 0.196*** | 0.0746*** | 0.184*** | -0.0859*** | 1 |  |  |
| eversexwork (=1) | -0.00431 | -0.00612 | -0.00813 | -0.00267 | -0.00248 | -0.00302 | -0.129*** | 0.183*** | 0.144*** | 0.126*** | 0.0801*** | -0.211*** | 0.315*** | 1 |  |
| livegeo | 0.00216 | -0.0113 | -0.00197 | -0.0107 | 0.00211 | -0.00260 | -0.0440*** | 0.103*** | 0.118*** | 0.0385*** | 0.0828*** | -0.0974*** | 0.0396*** | 0.0709*** | 1 |

Significant at *p < 0.05, ** p < 0.01, *** p < 0.001

LA-PrEP: long acting injectable pre-exposure prophylaxis

# **References for Supplementary Material**

1. Ghijben, P., Lancsar, E. & Zavarsek, S. Preferences for Oral Anticoagulants in Atrial Fibrillation: a Best–Best Discrete Choice Experiment. PharmacoEconomics 32, 1115–1127 (2014). <https://doi.org/10.1007/s40273-014-0188-0>
2. Galárraga O, Kuo C, Mtukushe B, Maughan-Brown B, Harrison A, Hoare J. iSAY (incentives for South African youth): Stated preferences of young people living with HIV. Soc Sci Med. 2020 Nov;265:113333. doi: 10.1016/j.socscimed.2020.113333. Epub 2020 Aug 29. PMID: 32896799; PMCID: PMC7738405.
3. StataCorp. 2023. cmxtmixlogit — Panel-data mixed logit choice model. College Station, TX, USA. https://www.stata.com/manuals/cmcmxtmixlogit.pdf
